# Supplementary figures and images for: Synthesis of Xylan-Click-Quaternized Chitosan via Click Chemistry and Its Application in the Preparation of Nanometal Materials
Source: Molecules. 2022 May 27;27(11):3455. doi: 10.3390/molecules27113455 (PMC9182352; doi:10.3390/molecules27113455)

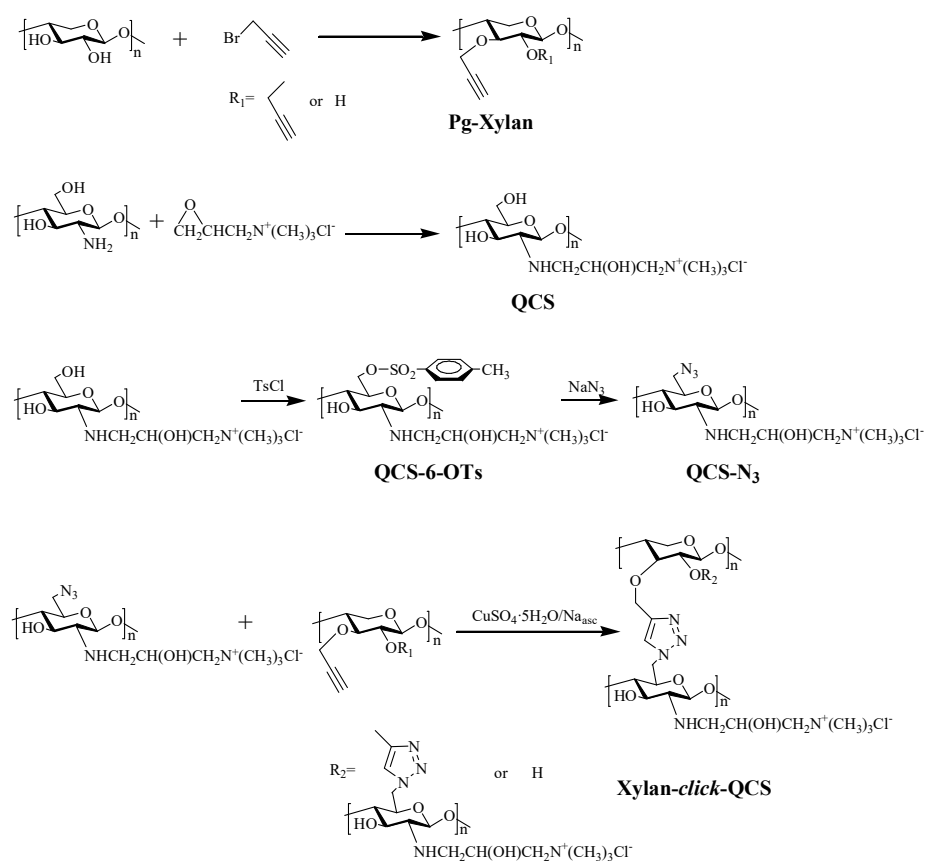

**Figure S1.** The synthesis process of xylan-click-QCS.

Supplement: Supplementary file 1 [file molecules-27-03455-s001.zip › molecules-1719710-supplementary.pdf]
